# Application of electro oxidation process for treating wastewater from petrochemical with mixed metal oxide electrode

**DOI:** 10.1038/s41598-024-52201-5

**Published:** 2024-01-19

**Authors:** Amir Mirshafiee, Mohammad Nourollahi, Alireza Shahriary

**Affiliations:** 1 Health Research Center, Life Style Institute, Baqiyatallah University of Medical Sciences, Tehran, Iran; 2 Department of Environmental Health Engineering, Faculty of Health, Baqiyatallah University of Medical Sciences, Tehran, Iran; 3 Chemical Injuries Research Center, Systems Biology and Poisonings Institute, Baqiyatallah University of Medical Sciences, Tehran, Iran

**Keywords:** Environmental sciences, Chemistry, Energy science and technology, Engineering

## Abstract

Petrochemicals require a large volume of water for their operation, which results in the production of a large volume of wastewater. Treatment of petrochemical wastewater is an important process before discharging it into the environment. This research examines the treatment of real petrochemical wastewater using the electrochemical oxidation process. Direct anodic oxidation is an effective advanced electrochemical oxidation process (AEOP), with different electrodes using a parallel plate electrochemical reactor. Four types of real wastewater were received from different petrochemical units were treated by AEOP. Real wastewater samples with chemical oxygen demand (COD) concentrations ranging from 20,450 to 52,300 mg/l. The main goal of this research is to make electrodes of Mixed Metal Oxide (MMO), which can reduce the treatment time and electricity consumption for oxidation, greater stability of the surface of the electrodes. Investigation of the rate constant kinetics shows that high COD removal efficiency can be achieved following the pseudo-second order reaction rate (R^2^ > 98%). When the wastewater pH is less than 5, COD removal efficiency is higher and the treatment process will be successful, which succeeded in removing 79% COD, but in alkaline wastewater, COD reduction efficiency was not satisfactory. The electricity consumption for 79% removal during 6 min was 117 kWh/m^3^. As a result, due to the very short time of the process (6 min), it can be used as one of the pre-treatment steps of petrochemical wastewater with acidic pH.

## Introduction

One of the most important environmental issues in large industries is wastewater treatment. In industrial wastewater, due to the materials in it, the usual methods such as activated sludge are not efficient, and the aeration process will be time-consuming and expensive. Advanced oxidation methods have been highly regarded in the treatment of biologically untreatable pollutants. The common methods of advanced oxidation include the use of oxidants such as hydrogen peroxide, ozone, and ultraviolet light and their combination. Considering the high costs of these methods for treatment and the difficulty of transferring these compounds to the desired location, other methods are suggested. One of the advanced oxidation methods is the electrooxidation method, which performs the treatment process by applying electric current, and the amount of sludge is much less than other wastewater treatment methods.

Wastewater from oil refinery and petrochemical industries is hazardous due to its high COD, phenols, benzene, heavy metals, suspended solids, surfactants, corrosion inhibitors, etc. As a result, this wastewater must be treated before discharge or reuse. Conventional treatment methods are based on mechanical and physical–chemical methods such as separation and coagulation followed by biological treatment. These methods have shown various operational problems such as the production of toxic gases, production of residual sludge, and incomplete removal of refractory compounds. Many other innovative technologies have been studied to treat this type of wastewater as an alternative to physico-chemical and bioremediation processes to meet the limits of reuse and discharge. In the meantime, various electrochemical technologies have been proposed as an efficient alternative for the treatment of this type of wastewater. They include electroflotation (ECF), electrocoagulation (EC), direct and indirect anodic oxidation (AO), and electrofenton (EF)^1,2^.

Anodic oxidation is the most popular electrochemical technology for removing organic pollutants from wastewater. As a result, many studies have been published on the application of AO for the refining of petroleum and petrochemical industries (Table 1). In particular, due to the presence of high concentrations of NaCl in refinery wastewater, free chlorine or chlorine-oxygen species can be generated on the anode surface. These chemicals can oxidize organic pollutants until complete mineralization, which is the process of converting organic matter into inorganic matter such as carbon dioxide and water.

**Table 1:** Some research of electrochemical treatment of wastewater from oil refinery and petrochemical industries.

Wastewater characteristics	Process	Electrodes	Comment	References
TDS 34,030 mg dm^−3^ COD 5800 mg dm^−3^ BOD 4590 mg dm^−3^	EC + AO	Active metal-graphite-iron	EC and indirect AO COD and BOD reduced by over 90%, SS by 99%	^3^
Petrobras (Brazil) COD 4980 mg dm^−3^	AO	Ti/Ru_0.34_Ti_0.66_O_2_	57% of COD removal	^4^
25–30 mg dm^−3^ of benzene, toluene, ethyl benzene, xylene 5 mg dm^−3^ of phenol	AO	RuO_2_-TiO_2_-SnO_2_	Complete depollution in 0.5–2.5 h	^5^
COD 19,842 mg dm^−3^ BODS 475 mg dm^−3^ Phenol 59 mg dm^−3^ Oil and greases 56.7 g dm^−3^	AO	PbO_2_, Boron Doped Diamond (BDD)	COD removal of 85% and 96% with PbO_2_and BDD	^6^
TDS 4.38 g dm^−3^ Phenol2.7 mg dm^−3^ Oils and greases 15 mg dm^−3^ COD 1588 mg dm^−3^	AO	Pt and BDD, tank cell	COD removal of 85% and 98% using Pt and BDD energy consumption: 109 and 140 kWh/dm^−3^ for BDD and Pt anodes	^7^
COD 4753 mg dm^−3^ NH3-N 3308.7 mg dm^−3^ \6latile phenol146 mg dm^−3^	AO + EF	Graphite + Fe particle	EC + Fe + Air + pH 3 COD value decreased to 479.4 mg dm^−3^ and NH_3_-N decreased to 17.48 mg dm^−3^	^8,9^
Phenol192.9 mg dm^−3^ COD 590 mg dm^−3^ Conductivity 15.63 rnS cm^−1^	AO EC, EF	BDD, RuO_2_ Fe electrode	Efficiency: EC < AO-RuO < AO-BDD < EF With EF Phenol removal of 98.74% and COD removal of 75.71%	^10^

Ramalho et al. studied the anodic oxidation of wastewater produced by oil exploration at a Petrobras plant in Brazil using a RuO_2_-TiO_2_-SnO_2_ anode. Under galvanostatic conditions, it was observed that the performance of the electrode material was influenced by the current rate. This fact indicates that hydrocarbons are removed by direct oxidation on the anode surface and by indirect oxidation with other generated oxidants, such as active chlorine formed by the oxidation of NaCl. Almost complete removal of pollutants was achieved after 2.5 h of electrolysis^5^.

The efficiency of the Ti/RuO_2_ anode for the treatment of Brazilian oil industry wastewater was investigated by Santos et al. The removal of COD after 120 min of electrolysis, with a current density of 10 mA/cm^2^, was about 96% for the wastewater. Complete removal of COD from both wastewaters was achieved when the current density was increased from 10 to 30 mA/cm^2^. The increase in current density also led to higher energy consumption^4^.

Gregory et al. studied the possibility of anodic oxidation for the removal of petroleum hydrocarbons from the wastewater produced by the oil exploration of the Petrobras plant in Tunisia using PbO_2_ and Boron Doped Diamond (BDD) electrodes. Compared to PbO_2_, BDD anode showed better performance in removing petroleum hydrocarbon compounds from produced water. COD removal using PbO_2_ and BDD was approximately 85% and 96% after 11 and 7 h. The average values of energy consumption at the end of electrochemical treatment using BDD and PbO_2_ electrodes were 38 and 46 kWh. These results show the high performance of anodic oxidation of BDD in petrochemical wastewater treatment^6^.

Rocha et al. investigated the anodic oxidation of wastewater using platinum (Ti/Pt) and BDD anodes in an electrolytic cell. They also evaluated the effect of several operational parameters such as flow, agitation rate, and temperature on COD removal and energy consumption. The removal of COD during the electrolysis of wastewater from the petrochemical industry at 25 °C and 30 mA/cm^2^ was only 46.5% and 57.5% for platinum and BDD. The electrochemical treatment of wastewater in the presence of 5 g of Na_2_SO_4_ at 60 °C temperature allows effective treatment of these organic compounds due to the high amounts of effective hydroxyl radicals and peroxydisulfates produced from water oxidation^11^.

Mirshafiee et al. conducted a study on the combined method of anaerobic treatment system in laboratory scale to remove oil from synthetic wastewater using a Biofilm Electrode Reactor (BER). The highest COD removal efficiency using the introduced method was 86.7%, and using general biological processes was 65.9%. In their research, the energy consumption of the biochemical system was 1.91 kWh/m^3^ (kilowatt hour per cubic meter)^12^.

Based on the results of Rocha et al.^11^, dos Santos et al.^7^ investigated a semi-industrial scale electrochemical flow system for petrochemical wastewater treatment using Titanium/Platinum (Ti/Pt) and BDD anodes. They showed that the research results were successful because compared to the results of Rocha and his colleagues, which were done in batch mode, a higher performance was obtained in the removal of organic matter. Even in the flow cell, the BDD anode has the ability to remove COD significantly faster than platinum due to its higher oxidation ability with the high reactivity of strong oxidant species (hydroxyl radicals and peroxydisulfates) produced on this electrode. Under suitable operating conditions, COD of petrochemical wastewater was reduced from 2746 to 200 mg/l within 5 h with energy consumption of 56.2 kWh/m^3^ with BDD anode. These results showed that this system has a high efficiency in petrochemical wastewater treatment.

Yavuz et al. compared different electrochemical processes for the treatment of oil refinery wastewater, including anodic oxidation using a metal oxide electrode, BDD, mixed ruthenium, electrofenton, and electrocoagulation using an iron electrode. The complete removal of phenol and COD was achieved in almost all electrochemical methods except electrocoagulation, provided that the electrolysis time was longer. The most efficient method was the electrofenton process followed by electrochemical oxidation using BDD anode. In the electrofenton method, phenol removal was 98.74% in 6 min of electrolysis and COD removal was 75% after 9 min of electrolysis^10^.

**Figure 1:**
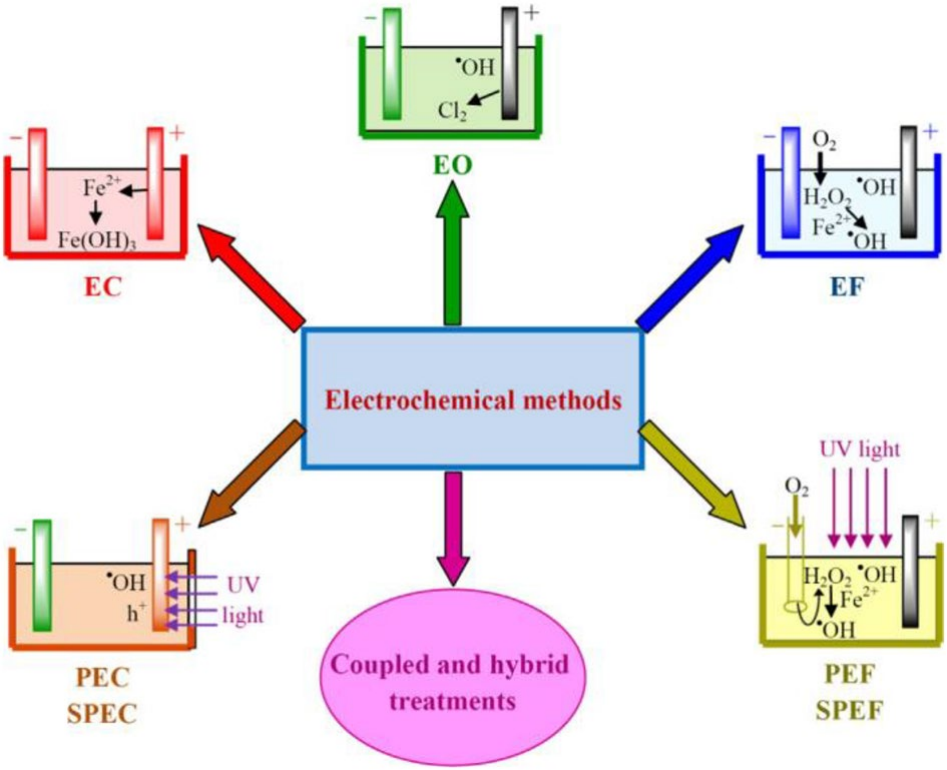
The most important electrochemical technologies used to remove organic substances from water and wastewater^13^.

The most important electrochemical technologies used to remove organic substances from water and wastewater are shown in Fig. 1.

Initially, the pioneering studies of Dabrowski in the 1970s, Kirk, Stuckey, Kutz, Chettiar, and Watkinson in the 1980s, and Johnson, De Battisti, and Kamnenellis in the 1990s helped clarify the principles of Electro-Oxidation (EO) for its application as an efficient alternative treatment method. After that, several research groups studied and researched the feasibility of EO for wastewater decontamination^13^. In later studies, a mechanism for oxidizing organic pollutants was proposed by Feng and Johnson^14^ based on the mediator function of reactive oxygen species formed at high potential as oxygen evolution reaction (OER) mediators. This process includes the transfer of anodic oxygen from H_2_O to organic materials through the absorbed hydroxyl radicals.

Mirshafiee et al. treated trinitrotoluene (TNT) from aqueous solution by electrocoagulation process with persulfate salt. They found that the electrocoagulation method can be effective as a practical method in reducing TNT from aqueous solution^15^.

As shown in Fig. 1, one of the methods of EO is the use of ultraviolet light. Karimi et al. and Azizi et al. investigated simultaneous removal of an organic matter and an inorganic by UV irradiation. They found that produces various radicals, including sulfides, carboxyl, hydroxyl, hydrated electrons, and various organic radicals that are highly reactive and help to precipitation inorganic substance^16,17^.

High efficiency, easy operating conditions, ease of automation, adaptability and low cost, especially when renewable energy is provided from wind and solar sources, are some of the most important advantages of EO technology^18^. For this reason, several groups have tested a wide variety of synthetic wastewater containing a variety of target compounds to understand the effects, operating conditions, disadvantages and potential efficiency of the EO approach^2,19^. The role of operating and operating parameters for the efficient use of EO approach strongly affects the type of wastewater and its constituents. For this reason, its industrial application is still limited. The advantages and disadvantages are summarized in Table 2^18^.

**Table 2:** The main characteristics of electro-oxidation technology^18^.

Main features of EO technology	
Ability	Production of active oxygen species (OH radicals), production of strong oxidants from salts, inactivation of microorganisms, versatility of evolution from two-dimensional to three-dimensional electrodes, without pH limitation and effective treatment of various organic pollutants in wastewater: (1) chemical industry, pulp industry and paper, petrochemical industries, pharmaceutical industries, textile industries, leather industries, food industries, landfill leachates; (2) agricultural industries (olive oil and dairy fertilizers) and (3) municipal and domestic sewage
Advantages	Versatility to treat large volumes, ease of automation, addition of non-toxic reagents to increase conductivity, use as pre-treatment or post-treatment of wastewater in combination with other technologies: biological treatment, Fenton oxidation, ion exchange, membrane filtration, membrane bioreactors and Electrochemical technologies
Disadvantages/challenges	Potential formation of halogen by-products, electrode deposition and corrosion phenomena, high operating costs due to high energy consumption, low wastewater conductivity, optimization of reactor hydrodynamic conditions and expensive electrodes

One of the most important parameters in electrochemical studies and applications is the constituent materials of the electrode, which can significantly affect the mechanism and the anodic reaction. The results of the studies in which different electrodes were used by other researchers are presented in this research and compared with the MMO coated electrode in this research.

Ruthenium oxide (RuO_2_) is an important catalyst in industry because it is widely used to produce Cl_2_ and O_2_. RuO_2_ has high conductivity and high thermal stability at temperatures above 800 °C. Ruthenium and ruthenium oxide also have high chemical stability^19^.

When oxide anodes (MOx) such as Ru-MMO are used, hydroxyl radicals (OH⋅), strong non-selective oxidizing agents, are produced. The mechanisms of oxidation of organic substances and the formation of hydroxyl radicals on the oxide anode are shown below (Eq. 1)^20^.

1 $${\text{MO}}_{{\text{x}}} + {\text{H}}_{2} {\text{O}}\mathop{\longrightarrow}\limits^{{k_{{{\text{OH}}}} }}{\text{MO}}_{{\text{x}}} \left( {{\text{OH}}^{*} } \right) + {\text{H}}^{ + } + {\text{e}}^{ - }$$

Hydroxyl radicals are absorbed on the surface of the electrode and decomposition of organic substances occurs according to Eq. 2.

2 $${\text{R}} + {\text{MO}}_{{\text{x}}} \left( {{\text{OH}}^{*} } \right)_{z} \mathop{\longrightarrow}\limits^{{k_{{\text{c}}} }}z/2 \cdot {\text{O}}_{2} + {\text{zH}}^{ + } + {\text{ze}}^{ - } + {\text{MO}}_{{\text{x}}}$$

BDD has a high potential for electrochemical applications in drinking water and wastewater treatment due to its extreme chemical reactivity and the possibility of using these electrodes (anode and cathode) in very acidic and alkaline environments^21^. When BDD anodes are used, they have the possibility of direct production of hydroxyl radicals (OH⋅) from water electrolysis with very high current efficiency (with amperes or high voltage) according to Eq. (3)^22^.

3 $${{\text{H}}}_{2}{\text{O}}\to {{\text{OH}}}^{*}+{{\text{H}}}^{+}+{{\text{e}}}^{-}$$

In this research, the feasibility of treating four real petrochemical wastewater samples from different production units using the EO method has been investigated. The performance of this electrochemical method was also evaluated and compared with the results of other researchers. The main goal of this research is to make electrodes made of MMO, which, due to obtaining a combination of metal oxides, can reduce the treatment time and electricity consumption for oxidation and greater stability of the surface of the electrodes (increasing the lifespan), the efficiency of the process. Also, investigating the changes in real petrochemical wastewater samples pH on the treatment efficiency by developed electrodes.

The objectives of this research are the development of MMO electrodes in order to achieve the following:

1. reduce the treatment time
2. reduce electricity consumption for oxidation
3. more stability of the surface of the electrodes (increasing the lifespan electrodes)
4. Investigating the changes in wastewater pH on the treatment efficiency by developed electrodes.

## Materials and methods

In Karun Petrochemical, all kinds of basic and main petrochemical products are supplied to the domestic and global markets using chlorine, carbon monoxide and hydrogen gases, as well as toluene, benzene, formalin, ammonia, nitric acid and sulfuric acid.

Four types of wastewater from different petrochemical units that produce isocyanates, including Toluene diisocyanate (TDI) and Methylene diphenyl diisocyanate (MDI) and complete the value chain of polyurethanes, have been received to be purified by the EO method and using coated electrodes. All studies and experiments were performed at ambient temperature (20–25 °C) with a volume of wastewater of 300 ml. The specifications of the petrochemical wastewater used are summarized in Table 3.

**Table 3:** Characteristics of the real wastewater sample of Karun petrochemical.

Phase	No	TDS (mg/l)	pH	COD (mg/l)
1	1	83,049	5	44,650
	2	5110	8	26,900
2	3	41,922	11	20,450
	4	87,801	14	52,300

Real wastewater is produced in two separate phases and each phase has two types of wastewater. The characteristics of four different types of wastewater related to phase one and two of Karun Petrochemical are shown in Table 3.

The composition of wastewater (1) includes mononitrotoluene, dinitrotoluene, nitric acid and water. This wastewater is the result of the condensation of steam coming out of the nitric acid concentration unit and the wastewater of the toluene nitration process.

Wastewater compounds (2) include Meta toluene diamine, mononitrotoluene, orthotoluene diamine, toluidine, nitrocrosols, sodium bicarbonate compounds, sodium chloride, nitrophenols and water. This wastewater is the result of washing Dinitrotoluene) DNT (with nitric acid, which is from the scrubber related to the output of phase one.

Wastewater compounds (3) include methyl diamine, cyclohexanone, tar aniline, aniline, ammonia and water. This wastewater is the result of the process of separating aniline from other organic substances and nitrobenzene, and the flow containing aniline is the wastewater.

Wastewater compounds (4) include aniline, methanol, cyclohexanone, methyl diamine, sodium carbonate, sodium chloride, sodium hydroxide, ortho dichlorobenzene, carbonyl dichloride, carbon monoxide, carbon dioxide, methyl diphenyl isocyanate and phenyl iso Cyanate. This wastewater is the output of the second phase to the caustic scrubber.

In Table 3, the wastewater has a high concentration of total dissolved solids ranging from 5110 to 87,801 mg/l. COD ranges from 20,450 to 52,300 mg/l pH ranges from 5 to 14.

### Experimental procedure

In this research, the electrochemical oxidation of wastewater was carried out using a Ruthenium-Iridium Mixed Metal Oxide (Ru-MMO) electrode in a parallel plate reactor with a volume of 300 ml. Two anodes and three cathodes made of Ru-MMO in dimensions of 12 cm × 27 cm were prepared and installed in the reactor. In the tests, the current intensity was 64 amps. Experiments were performed in batch mode and the wastewater was circulated by a pump. This research was done without supporting electrolyte.

### Analytical procedures

COD was measured using a COD reactor (HACH) and a spectrophotometer. All COD analyzes were performed in duplicate and average values are reported. The samples were analyzed using a spectrophotometer (Rayleigh, Vis-7220 / UV-9200).

COD is usually used as an analytical method to evaluate the treatment of pollutants in wastewater. The following equation was used to determine the removal efficiency (Re%):

4 $$\mathrm{Removal \;Efficiency }=\frac{{C}_{0}-{C}_{t}}{{C}_{0}}\times 100$$

where C_0_ represents the initial concentration (mg/l) and C_t_ represents the concentration at time t in terms of mg/l.

A scanning electron microscope (SEM) from Seron technology with model number AIS-2100 was used to study the surface of the electrodes. Stateron 3234.4 and 3234.9 power supplies, OGSM3900 digital multimeter, MultifixMC 1000 PEC peristaltic pump, Orion 420 A pH meter were used as auxiliary equipment in the experiments.

### Kinetic study

Determination of reaction rate is an important aspect in electrochemical studies. A kinetic study was carried out to outline the details of the pollutant removal reaction. Kinetic studies can help to design, model and implement the process on a practical and industrial scale^23^.

Kinetic studies were carried out using different applied currents at ambient temperature and constant volume of wastewater. First and second order relationships were used to evaluate the kinetic reactions. Equations (5) and (6) correspond to the first order and Eq. (7) corresponds to the second order.

5 $$-\frac{d}{dt}\left[C\right]=K\left[C\right]$$

6 $$ln\frac{\left[{C}_{t}\right]}{\left[{C}_{0}\right]}=-K.t$$

7 $$\frac{1}{{C}_{t}}-\frac{1}{{C}_{0}}=-{K}{\prime}.t$$

where, k represents the constant rate of the pseudo-first order model, and k’ represents the constant rate of the pseudo-second order model^24^.

### Energy consumption

In the experiment, the decontamination or treatment of petrochemical wastes is checked to reduce their COD. Energy consumption (EC) per treated wastewater volume was estimated and expressed in kilowatt-hours per cubic meter. The average cell voltage, during electrolysis, is considered to calculate the energy EC as follows^11^

8 $$\mathrm{Energy \;Consumption }= (\frac{\Delta Ec \times I \times t}{3600 \times V})$$

where, t is the electrolysis time (s); ΔEc is the average cell voltage (V) and I is the electrolysis current intensity in amperes (A) and V is the sample volume in cubic meters^11^.

**Figure 2:**
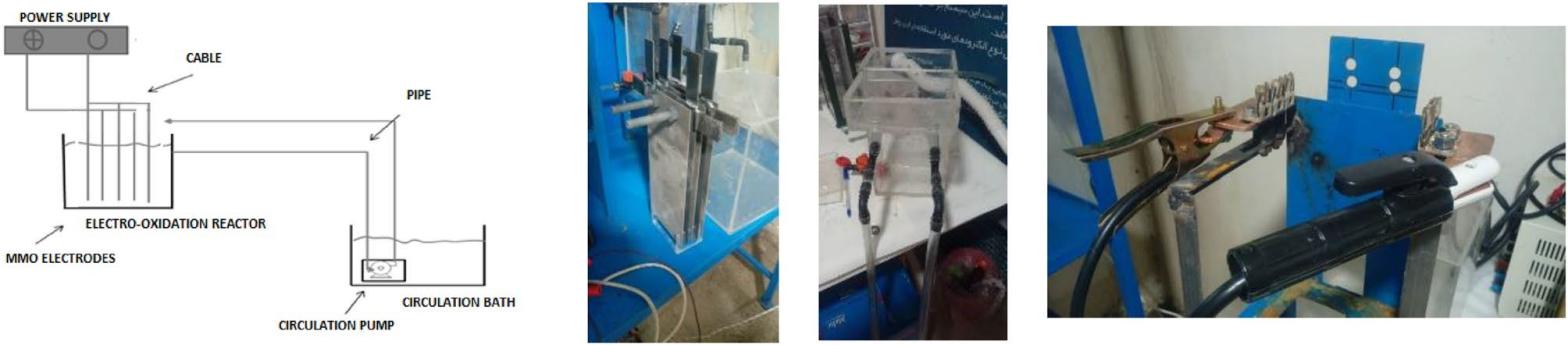
Schematic of electroco-oxidation process setup.

The schematic of electroco-oxidation process is shown as Fig. 2.

### Factors affecting anode performance

The performance of an electrode is rated in terms of its ability to treat organic substances with minimal energy and time consumption. The nature of the electrode, applied current density, duration of electrolysis, supporting electrolyte, pH, temperature and cell geometry significantly contribute to the efficiency and energy consumption of the electrochemical oxidation process. The nature of the electrode depends on its manufacturing technique, composition and structure^25^.

### Construction technique

Physical characteristics such as surface morphology, thickness of metal oxide layer, composition, dimensions and microstructure and chemical properties such as availability of higher oxidation state, its affinity for hydroxyl radicals, stability at different pH levels and active species produced in the presence of different electrolytes as well as wear properties of the anode affect its ability to oxidize organic materials (electrochemical activity) or produce active oxidants (electrocatalytic activity). The manufacturing technique can modify the chemical and physical nature of the coating and, as a result, its electrocatalytic and electrochemical activities. The key to creating a strong adhesion of the metal oxide coating on the substrate surface is the process and selection of materials with appropriate composition^26^.

### Surface layer and bottom layer

**Figure 3:**
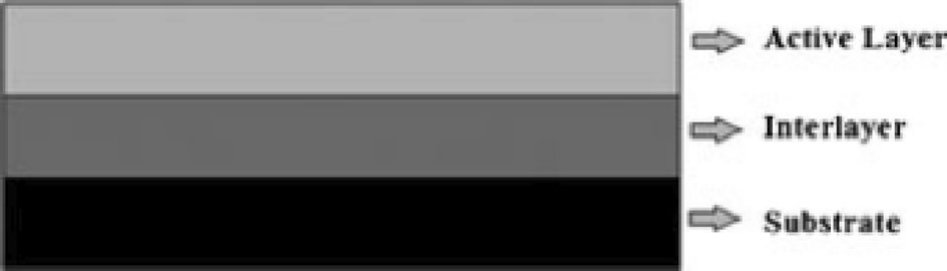
Multilayer substrate coated with metal oxide^26^.

The oxygen formed on the surface of the anode moves through the metal oxide coating and deactivates the substrate metal. This process is eliminated by including a surface layer that connects the active metal oxide coating and the substrate. Chemical stability, corrosion resistance and useful life of the anode are increased by placing an intermediate layer^26^. A bottom layer of stable metal oxide is applied on the substrate and then an outer active layer is placed on it. As shown in Fig. 3, it is represented as the substrate/interlayer/active surface layer. The middle layer supports the active metal oxide layer for strong adhesion to the substrate and helps to protect the stability and increase the electrocatalytic activity of the electrode. It is very important that the middle layer has high conductivity and is not passive in terms of process.

**Figure 4:**
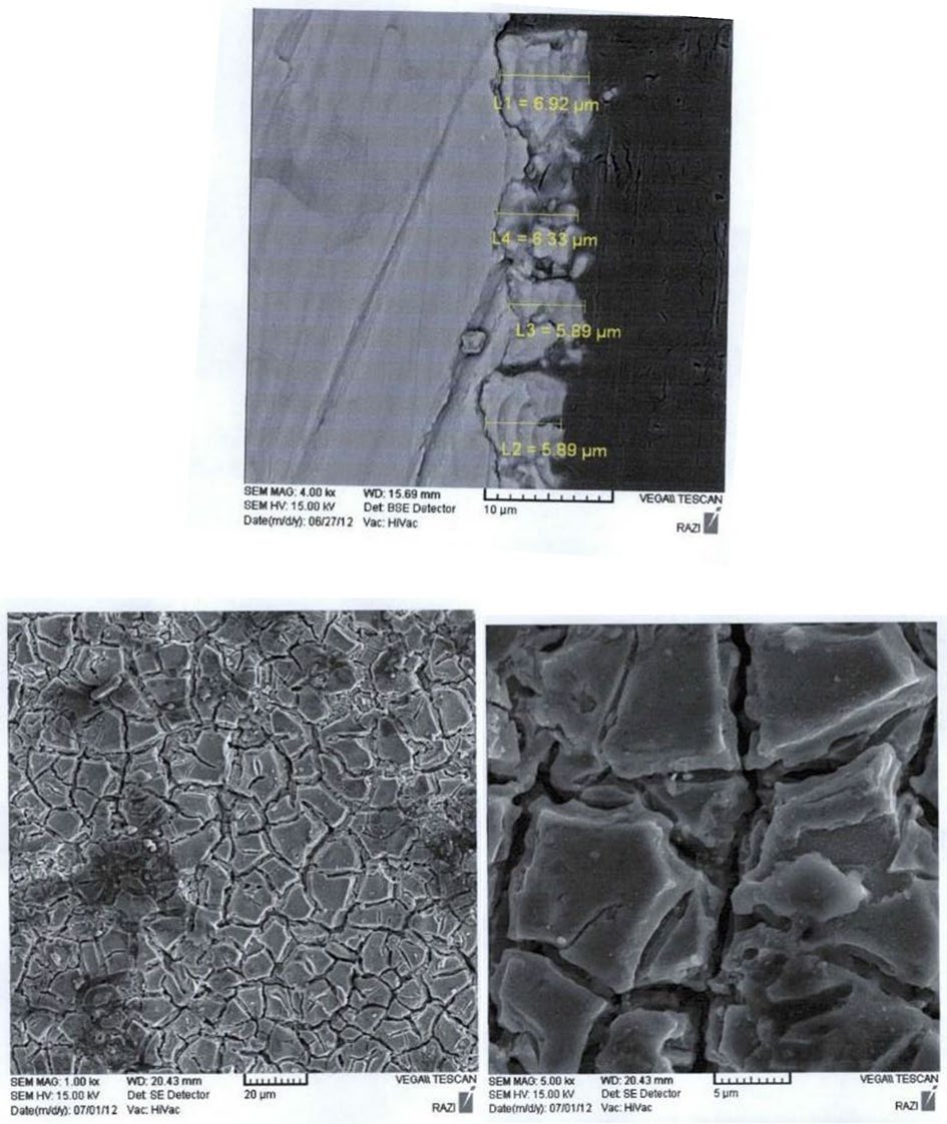
SEM images of the anode electrode surface and its coated amount.

The main goal in this research is to make electrodes made of MMO, which can be achieved by reducing treatment and if possible reducing electricity consumption for oxidation while increasing surface stability (increasing lifetime) which increases efficiency of EO process. For this purpose, titanium was used as a base for making the anode electrode and Titanium Dioxide (TiO_2_), Iridium Dioxide (IrO_2_) and Ruthenium Dioxide (RuO_2_) with different percentages were used to cover its surface. The thickness of the coating used on the anode electrode in these tests was up to 8 µm. SEM images of the anode electrode surface and its coated amount is shown Fig. 4.

**Figure 5:**
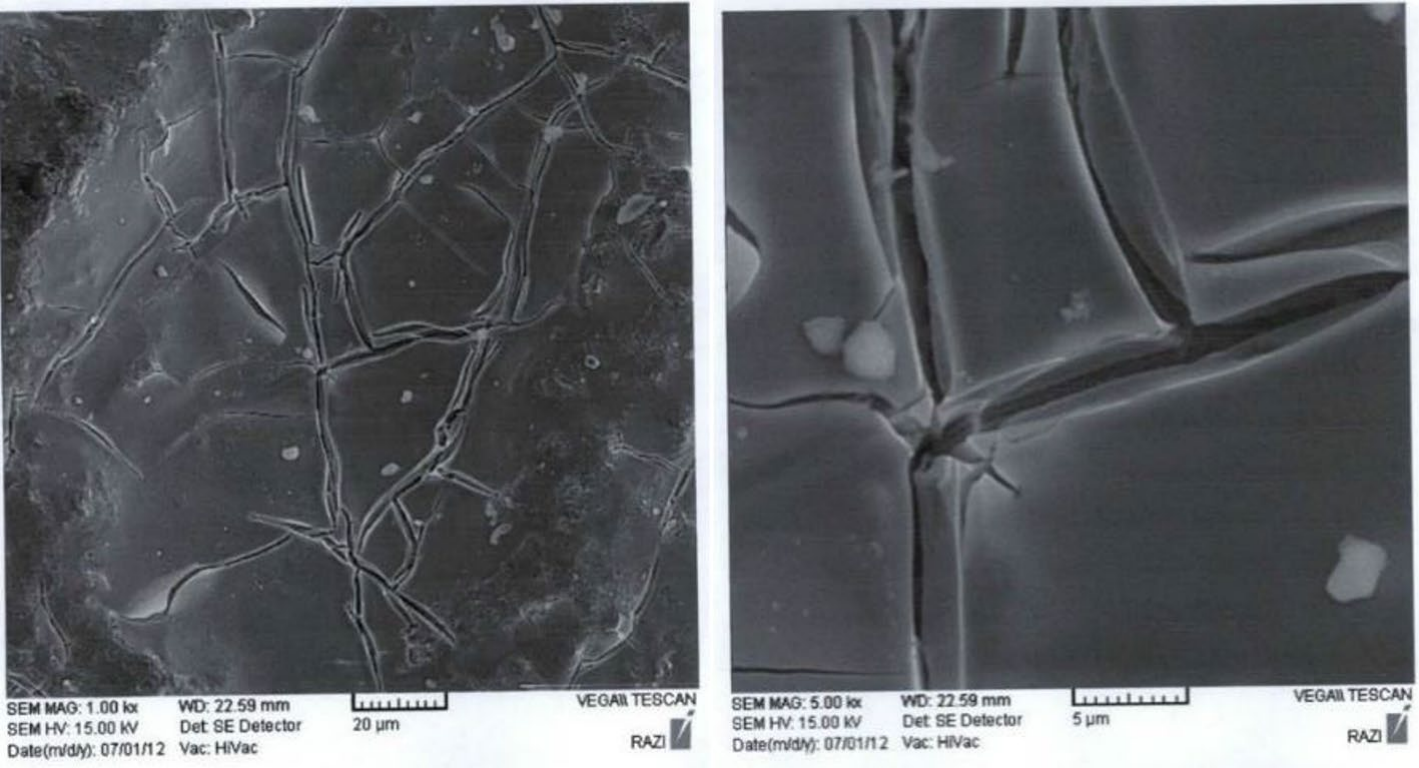
SEM images of the cathode electrode surface and its coated amount.

For the cathode electrode, nickel metal was used as a base and Al_2_O_3_, NiO, RuO_2_ and IrO_2_ with different percentages were used to cover its surface. The thickness of the coating used on the cathode electrode in these experiments was up to 8 µm. SEM images of the cathode electrode surface and its coated amount is shown in Fig. 5.

### Ethical approval

This research was approved by the Ethics Committee, Environmental Health Research Committee of Baqiyatallah University of Medical Sciences (IR.BMSU.BAQ.REC.1400.081) and project number # 400000235.

## Results and discussion

After 6 min of electrolysis in the reactor using the electrooxidation process, for wastewater No. 1, 2, 3 and 4 whose compositions were mentioned earlier, the percentage of COD removal was 79.1%, 54.56%, 57.3% and 32.2%. As it can be seen, the percentage of COD removal for different wastewater is very different, which will be discussed in the following.

### Effect of pH value

It seems that pH is one of the main factors affecting the reaction rate of wastewater treatment. In general, it can be seen that the more acidic the wastewater, the better the COD removal efficiency. It is clear that COD removal efficiency decreases significantly with increasing pH value. In other words, wastewater number 4 with a pH of 14 had the lowest COD removal efficiency in the tests. The results of Kumar et al. in the treatment of mononitrotoluene (MNT) by electrochemical method also showed that the percentage of treatment efficiency increased from pH 2–5 and decreased from pH 5–10. In such a way that the highest percentage of treatment efficiency occurred at a pH of about 5^27–29^. Mirshafiee et al. also showed using the electrochemical method that COD removal efficiency was higher when the tested solution was at acidic pH, which was consistent with the results obtained in this research^17^.

Chen and Zhu treated dinitrotoluene (DNT) in wastewater using persulfate anions combined with ultrasonic irradiation. They showed temporal patterns of total organic carbon (TOC) removal percentage as a function of pH values and found that treatment efficiency of nitrotoluenes decreased significantly with increasing pH values^30^.

Yu et al. investigated electrochemically activated persulfate for diuron degradation and reported that activation of sulfate radicals decreased when initial pH reached alkaline conditions. Regeneration of doubly positive iron ions from ferric ions in cathode was also eliminated and reduced degradation rate of diuron. It can be concluded that electrochemical process is more effectively used in acidic conditions compared to alkaline conditions^31^.

according to results obtained in this research and results of other researchers that have been mentioned, it can be found that rate of production of radicals to reduce COD in wastewater can be increased in acidic solutions compared to alkaline solutions. Of course, it should be noted that increase in acidity should be done according to coating done on anode and cathode so as not to cause corrosion of coatings.

### Effect of electrolysis time

In this paper, the goal of treating different real petrochemical wastewater was not to completely remove COD. Rather, we sought to achieve a combination of materials for MMO electrodes so that an acceptable removal percentage can be achieved with a short time in the electrolysis process.

**Figure 6:**
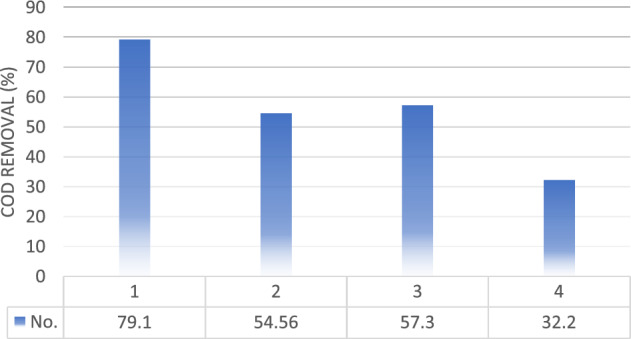
The final result of COD removal percentage on four different wastewater samples at the end of the electrooxidation process.

In Fig. 6, COD removal percentages for wastewater 1–4 were 79.1%, 54.56%, 57.3%, and 32.2%, using EO method. Table 4 also shows specifications such as average current, primary and secondary COD voltage.

**Table 4:** Results from experiment using electrooxidation treatment four sample wastewater.

No	Time (min)	Current (A)	Applied potential (V)	Initial COD (ppm)	Final COD (ppm)	Removal efficiency (%)
1	6	64	5.5	44,650	9332	79.1
2	6	64	4.1	26,900	12,223	54.56
3	6	64	4.1	20,450	8732	57.3
4	6	64	12	52,300	35,459	32.2

**Figure 7:**
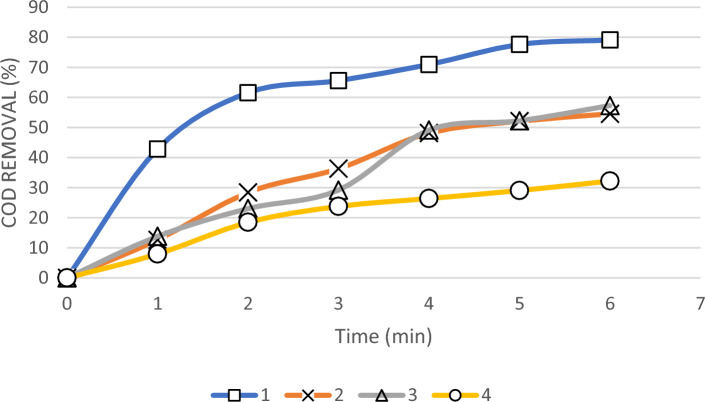
The effect of time on COD removal percentage on four different real petrochemical wastewater samples.

Figure 7 shows the change of COD reduction rate with electrolysis time for 4 types of real petrochemical wastewater, so that other variables were considered constant. As can be seen in Fig. 7, for wastewater No. (1), with the increase of the reaction time from the beginning of the experiment to the third minute, the percentage of COD removal has been carried out with a high slope and up to 65%, and after that the percentage of COD removal has not improved significantly. COD removal percentage can be related to changes in concentration and pollutants in this wastewater by electrochemical treatment.

For wastewater No. (2) and (4), the process of COD reduction was almost the same, with the difference that the slope of the graphs was slightly different. For wastewater No. (3), the COD reduction trend from the third to the fourth minute had a significant increasing slope. This slope change may be due to the pH change in the wastewater during the reaction due to the presence of cyclohexanone. As it was concluded earlier, the production rate of radicals increases to reduce COD in the wastewater, in an acidic environment.

Kumar et al.^27^ used two methods of electrofenton and electroperoxide to remove MNT and showed that the electrofenton reaction is relatively faster than the electroperoxide reaction. During the MNT removal test by electroperoxide method, the solution was observed to be turbid due to the formation of some intermediates during turbidity decomposition. They concluded that the electrofenton process is superior to the electroperoxide process in terms of rapid treatment time of MNT from 50 to 1 ppm within 2 h.

In other researchers’ results (Kumar et al., Babupunosamy et al. and Umar et al.)^27–29^, their experiment duration was at least 120 min. This is despite the fact that in this research, we have greatly reduced this duration which is an achievement in terms of purification time. In other words, since wastewater No. (1) has COD equal to 44,650 mg/l, and after only 6 min of electrolysis, 79% COD removal efficiency was obtained.

### Effect of applied current

The electric potential difference and the intensity of the current applied to the electrode are also important parameters related to MNT wastewater treatment for the EO process. To study the effect of applied voltage on MNT treatment, the voltage was changed from 4 to 12 V without interfering in the purification process and keeping the temperature of the laboratory environment constant. This voltage change was applied if the current intensity was constant at 64 amperes during the experiments. The current intensity is applied between fixed MMO electrodes and the electrodes are kept at a distance of 3 mm from each other immersed in the solution.

In Kumar’s research^27^ it has been observed that at a voltage lower than 6 V, the treatment of MNT is very slow, which is due to the insufficient formation of OH radicals during the chemical reaction. At voltages above 18 V, turbidity was observed, which may be due to the formation of complex ionic compounds. According to their results, the MNT purification efficiency has decreased in both cases, that is, when voltage is less than 6 V and more than 18 V. Also, in research results of Chen and Zhou^30^, Mirshafiee et al.^15^, it was seen that in treatment using electrochemical method, with increase in current density due to increase in voltage or intensity of current, efficiency of treatment has also increased.

By comparing range of voltage change in this research and results of Kumar et al.^27^ it can be seen that results obtained in voltage change in this study were consistent with their results.

The best COD removal conditions were obtained with a distance of 3 mm between electrodes. In Kumar’s research^27^, distance between electrodes was at a distance of 40 mm which were kept immersed in solution. Meanwhile, according to repetition of experiments in this research, optimal distance for placing electrodes was found to be 3 mm. This interval was partially obtained by repeating experiments where best COD reduction efficiency occurs. At electrode distances less than 3 mm, gas phase formed on electrodes has reduced speed of reactions and contact of radicals with pollutants. Also, when electrodes are more than 3 mm apart, there is an increase in ohmic resistance between electrodes and as a result decrease in current.

In this study, applied voltage for efficient purification of MNT was considered from 4 to 12 V for electrochemical process. Current intensity during tests was constant at 64 amperes.

Electricity consumption for six minutes of analysis time is calculated by averaging voltage and current. The estimation of treatment costs is very important; Table 5 reports the electricity consumption in (Kwh/m^3^) after 6 min of electrochemical process.

**Table 5:** Summary energy consumption of treatment process at 6 min.

No	Applied potential (v)	Current (A)	Time (min)	Volume (l)	Energy consumption (Kwh/m^3^)
1	5.5	64	6	0.3	117
2	4.0	64	6	0.3	85
3	4.1	64	6	0.3	87
4	11	64	6	0.3	235

As can be seen in this study, the electricity consumption for wastewater No. 1, 2, 3 and 4 was equal to 117, 85, 87 and 235 kWh/m^3^. Wastewater No. 4 has relatively low COD removal efficiency due to high electricity consumption. This is due to the lack of proper performance of this process with the MMO electrode for alkaline wastewater, which was explained earlier and compared with the results of other researchers.

Nevertheless, the electrochemical treatment time can be significantly reduced by using this process with the MMO electrode for acidic wastewater because according to the coating performed in this study, after only 6 min, the removal efficiency is 79% achieved for wastewater No. 1. Dos Santos et al.^7^ results were conducted on a petrochemical wastewater with a COD equal to 2746 mg/l with BDD electrodes and found that after 5 h of treatment and consumption of 56.2 kWh/m^3^, the COD can be reduced to 200 mg liters decreased. Considering that the type of wastewater in this research is from petrochemical units but the COD of wastewater No.1 is equal to 44,650 mg/l.

Yavuz et al. compared different electrochemical processes for oil refinery wastewater treatment including anodic oxidation using metal oxide electrode, BDD, ruthenium mixture, electrofenton and electrocoagulation using iron electrode. Their result showed that the most efficient method was electrofenton process followed by electrochemical oxidation using BDD anode and then MMO electrodes^10^. This is despite the fact that in the electrofenton method it is necessary to inject hydrogen peroxide into the wastewater which is not a cost-effective method in case of continuous wastewater production and high volume of wastewater. The preparation and space required for hydrogen peroxide storage is also one of its disadvantages. BDD electrodes are still very expensive and their high electricity consumption cannot be used to treat a large volume of wastewater. If MMO electrodes still have a lower manufacturing cost compared to BDD electrodes they can be used for industrial wastewater treatment pilots.

By comparing the results obtained in this research and the results of Santos et al.^4^ and considering that they used the expensive BDD electrode, it can be seen that by changing the type of electrode and most importantly the type of its coating with MMO electrodes, acceptable removal efficiency percentages were reached in a short time. Also, as mentioned, the cost of making BDD electrodes is much higher than MMO electrodes.

### Kinetics study

**Figure 8:**
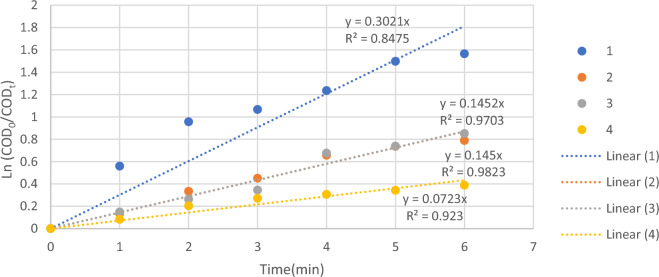
Kinetic diagrams of COD removal from petrochemical wastewater through electrochemical process: pseudo-first order.

Figure 8, the graph of ln (COD_0_/COD_t_) versus oxidation time shows a straight line with K_app_ slope. Anodic oxidation of petrochemical wastewater showed an exponential behavior with applied currents. The results of the present oxidation kinetics are consistent with the results observed in research (Elaoud et al., Panizza and Cerisola, Sopaj et al.)^32–34^. This behavior is often characteristic of oxidation under mass transfer control when the process is carried out by applying a current higher than the limiting current (i > i_lim_) with COD concentration decreasing according to an exponential trend^35^. pseudo-first order rate constant and pseudo-second order rate constant coefficient of COD removal kinetics is shown in Tables 6 and 7.

**Table 6:** Pseudo-first order rate constant coefficient of COD removal kinetics.

Pseudo-first order							
No	Time (min)						
	0	1	2	3	4	5	6
1	0	0.56	0.96	1.07	1.24	1.50	1.57
2	0	0.14	0.33	0.45	0.66	0.74	0.79
3	0	0.15	0.26	0.35	0.68	0.74	0.85
4	0	0.08	0.20	0.27	0.31	0.34	0.39

**Table 7:** Pseudo-second order rate constant coefficient of target COD kinetics.

Pseudo-second order							
No	Time (min)						
	0	1	2	3	4	5	6
1	0	0.000017	0.000036	0.000043	0.000055	0.000078	0.000085
2	0	0.000005	0.000015	0.000021	0.000035	0.000040	0.000045
3	0	0.000008	0.000015	0.000020	0.000047	0.000053	0.000066
4	0	0.000002	0.000004	0.000006	0.000007	0.000008	0.000009

**Figure 9:**
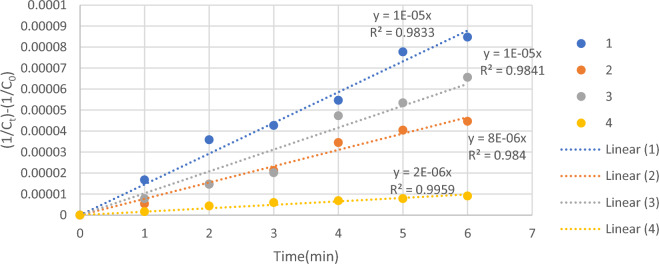
Kinetic diagrams of COD removal from petrochemical wastewater through electrochemical process: pseudo-second order.

For the experiments wastewater No. 3 and 4, the concentration of COD decreased linearly following a pseudo-first-order kinetic reaction. But for the experiment wastewater No. 1 and 2, the concentration of COD decreased linearly following a pseudo-second-order kinetic reaction which is shown in Figs. 8 and 9.

In the pseudo-first order model, the lowest rate constant in the electrochemical process was obtained with a numerical value of 0.072 for wastewater No. 4 and the highest rate constant was 0.3 for wastewater No. 1. In the pseudo-second order model, the lowest rate constant in the electrochemical process was obtained with a numerical value of 0.016 × 10^(−4)^ for wastewater No. 4 and the highest rate constant was 0.14 × 10^(−4)^ for wastewater No. 1. According to Table 8, the fastest reaction in the process took place for wastewater No. 1 among the real petrochemical wastewater studied.

**Table 8:** First-order and second-order rate constants for wastewater.

No	COD removal (%)	Pseudo-first order		Pseudo-second order	
		K (min^−1^)	R^2^	K × 10^(−4)^ (L/mg min)	R^2^
1	79.1	0.3	0.96	0.14	0.99
2	54.56	0.145	0.97	0.1	0.98
3	57.3	0.14	0.98	0.07	0.99
4	32.2	0.072	0.98	0.016	0.99

The design of pseudo-first order and pseudo-second order kinetic models for COD removal through the electrochemical process in different wastewater are shown in Figs. 8 and 9, and the results of the kinetic study are summarized in Table 8. For wastewater 1 to 4, due to the small difference of the correlation coefficient (R^2^) values for the pseudo-second-order kinetic model compared to the pseudo-first order kinetic model, it can be concluded that the COD removal reaction was better described by the second-order model.

The removal of COD is very complicated because petrochemical wastewater generally contains a variety of organic and inorganic pollutants, and a longer electrolysis time is required to remove COD compared to the decomposition of organic pollutants. Although the purpose of this study was to change the structure of the coated materials in the electrodes to reduce treatment time.

It is obvious that high pH solutions are less efficient for COD removal of petroleum and petrochemical wastewater because OH⋅ radical can be consumed by reacting with inorganic compounds in wastewater, including sulfate ions (${SO}_{4}^{-2}$) and bicarbonate ions (${HCO}^{-3}$) and carbonate (${CO}_{3}^{-2}$), which cause attraction. These reactions can significantly reduce efficiency of electrochemical process in oxidizing organic pollutants and reduce reaction rate constant.

## Conclusion

In this paper, real petrochemical wastewater was treated with the electrochemical method of anodic oxidation of MMO. For this purpose, electrochemical oxidation using MMO electrodes was used and the results of the experiments were analyzed with the results of other researchers. The treatment of refinery wastewater was investigated and evaluated with COD removal efficiency. The treatment of real petrochemical wastewater was investigated using parameters such as current density, pH of the solution and type of electrode material and reaction kinetics.

In general, according to the obtained results, when the pH of the solution is less than 5, COD removal efficiency is higher and the purification process will be efficient. The most efficient result was related to wastewater number 1, which succeeded in removing 79% COD. As a result, due to the very short time of the process, it can be used as one of the pre-treatment steps of petrochemical wastewater with acidic pH. By comparing the results obtained in this research with the results of other researchers, it can be seen that the electrochemical process followed by direct or indirect oxidation using MMO anode has a good efficiency in COD removal due to its very low manufacturing cost. It was also found that if the wastewater has alkaline properties, such as wastewater number 4, using this process to treat this type of wastewater is almost ineffective due to the very high consumption of electricity.

## Data Availability

The data that support the findings of this study are available from KARUN Petrochemical CO. but restrictions apply to the availability of these data, which were used under license for the current study, and so are not publicly available. Data are available from the authors upon reasonable request and with permission of KARUN Petrochemical CO.
